# The Necessity for Proper Care of School Children’s Eyes

**Published:** 1895-06

**Authors:** Dunbar Roy

**Affiliations:** Atlanta, Ga.; Professor Ophthalmology and Otology in Southern Medical College


### THE NECESSITY FOR PROPER CARE OF SCHOOL CHILDREN'S EYES.

By DUNBAR ROY, A.B., M.D.,

Professor Ophthalmology and Otology in Southern Medical College, Atlanta.

"An ounce of prevention is worth a pound of cure" is an old adage, yet the triteness of its meaning is appreciated in every domain of medicine. Hygiene and preventive medicine have in the last few years taken a strong hold upon the profession and public at large, and each day do we read publications from those who are their strongest advocates. That there is a reason for this every one must admit who keeps informed of the progress made in every department of medicine. Pathological anatomy and bacteriology have revealed to us the cause of many diseases which before remained obscure, and likewise in their revelations have they afforded us a more rational method of treatment. As we are thus better enabled to arrive at the root of every disease, so are we more capable of dispersing the germs of the same before they have been firmly implanted in the human system.

Why do we erect our quarantine stations, if it is not to keep our country free from the ravages of some exotic disease?

Why is it that the hygiene of our public places is now exciting such widespread and deep attention, if it is not through the fear that some lurking bacillus tuberculosis may claim an innocent victim as its own?

Why is it that the mother alone watches by the bedside of her little darling who is tossing amid the pain and fever of some infectious or contagious disease—some enemy of childhood, while all the rest of the world stands at a distance, if it is not through the fear that the same disease may implant its fangs upon some member of its own household?

Why then is all this care and solicitude, if it is not to keep the body in a healthy state for fear that it may be seized upon by some lurking malady?

Is not the proper regulation of our diet, the deglutition of our food, the attention to the calls of nature, absolutely necessary for the well-being of our bodies?

Every organ, strictly speaking, is a distinct entity, but its function to be properly executed requires the harmonious action of all; for the derangement of one impairs the usefulness of another. In the eyes of many the importance of any organ in the production of disease is largely dependent upon its size and the "current fad" of the medical age. How many of our ailments are often attributed by Southern practitioners to that one organ, the liver? How often is a woman made to believe that her whole organization consists solely of a nervous system and two ovaries? Alas, how often would the enthusiastic ophthalmologist make us believe that refractive errors and muscular anomalies are the most potent factors in the cause of every obscure nervous affection?

But in all extreme measures there is a germ of truth. There must be the proper supervision over every organ of our body if we wish the greatest good to accrue through the activity of the same; for unless the proper care be exercised then disease is very apt to follow.

If there is a tendency to cardiac hypertrophy, are we going to allow our patients to indulge in undue muscular exertion or stimulation? If there is a lack of the digestive ferments in the alimentary track, shall we allow our patient to partake of a Welsh rarebit every night before retiring? If there is a weakness of the vocal chords, are we to allow the inordinate use of the same?

With this preface before you, I wish to speak for a few moments in reference to the proper care of school children's eyes; to show you the necessity for the same, and in this manner make manifest to our Association the importance of the subject. Let me say in the outset that I am no extremist, no crank upon the importance of the eye in the causation of many reflex troubles, but there are certain ocular conditions which are frequently overlooked by the general practitioner, yet to the thoughtful ophthalmologist must be seen to exert a most decided influence upon the general health of the individual. The eye is a minute organ in comparison to the rest of the human frame, yet without it the very soul-life of our body were extinct! It is in relationship to our whole nervous system, and active as it is during every moment of our waking hours, one readily conjectures what an influence it could exert in many of our reflex troubles. It is only in the last few years that the attention of the profession has been prominently directed to the eye, as a cause for many of the nervous phenomena which arise in the human body, and to the importance of a thorough examination of the same to fortify them in their diagnosis. The subject is indeed important, and every day do we see the ophthalmic journals teeming with statistical reports upon the examination of school children's eyes, until now even the laity cannot feign ignorance of the matter.

That you may more readily understand the point to which I am alluding, I will state clearly the proposition which shall be discussed and which I wish to impress upon the medical profession of Georgia in order that at no late date steps may be taken for the proper rectification of the errors which shall be pointed out. My proposition is this: There are too many children in our schools between the ages of six and twenty who are using their eyes to the detriment of the same, in that there exists some refractive error, as far-sightedness, near-sightedness, or astigmatism, which should be corrected before any undue strain is put upon these visual members. These uncorrected errors produce many reflex disturbances in the body which are often attributed to some other cause, as digestive, uterine, or even to defective sanitary conditions of the schoolrooms. Not only are extraneous symptoms, as headaches, nervousness, etc., produced, but changes in the eye itself occur, which, if not noticed and corrected early, may go from bad to worse until there is a permanent injury to some portion of the globe.

The subject of the care of school children's eyes has been much more extensively studied in Europe, especially in Germany, than it has been in America; but of late our American confrères have been actively engaged in bringing it prominently before the profession of our own country.

As to what is the normal, or emmetropic, eye, is a point by no means settled among the best ophthalmologists. From numerous observations it has been found that the large majority of eyes of infants are hyperopic or far-sighted, and that the tendency with increasing years is rather in the direction of myopia or near-sightedness than in an increase in the hyperopia. For instance, Ely, in the examination of the eyes of 111 infants, found hyperopia in 69 per cent.,while Koenigstein, in Germany, in the examination of 281 infants, found hyperopia in 98 per cent. Since the above observations have been made numerous other examinations have in a large measure confirmed these statements. On the other hand, statistical observation among school children has revealed the fact that with increasing years, and therefore increased mental work, the tendency of the eyes is towards myopia, or near-sightedness, even passing into that refractive error from low degrees of hyperopia. Myopia is looked upon by some writers as a product of civilization. Such writers hold that among the aboriginal Indians and savages myopia is unknown, and that it is found more frequently as we ascend the scale of intelligence, or where there is a greater demand upon the eyes for close application. For instance, in Germany it is remarkable, and frequently commented upon, the number of people one sees wearing glasses, and nearly entirely for myopia, due in a large measure, as statistics show, to the increased ocular strain necessitated by the advancement in studies.

In the first place, is myopia, or near-sightedness, an abnormal condition of the eye? and if so, does it impair the real value of the visual member?

To consider for a moment that myopia is not recognized by you as an abnormal and pathological condition of the eye would be a reflection upon your intelligence; so, passing that by without further comment, let us consider the latter part of the proposition in regard to the impairment of its function when myopia exists. An eye that is the most comfort to its possessor is one which never occasions a cognizance of its presence. Not so with the myopic eye; for, besides interfering with the clear perception of the objects around us, which in itself is a great deprivation, it frequently causes pain and discomfort when at rest, and much more so when brought into activity. Besides, if the error be left uncorrected an increase of the myopia is sure to follow, and with that changes may occur in the interior of the eye which may finally lead to permanent inpairment of the sight.

This, I assure you, gentlemen, is no idle fancy, but a fact borne out by every-day clinical experience. A myopic eye is always a menace to its possessor, a circumstance alone which should lead to a proper correction of the error.

In the second place, if it can be shown by statistics that myopia increases among school children as they advance to higher grades, should not some measures be taken by which this increase may be minimized? The statistics of school children's eyes compiled in Germany, and also in America, are too familiar almost to warrant comment; but, for the sake of those who are not so conversant with them, I will mention some statistical reports as justifying the statements which have been made, and also to substantiate my second proposition.

Since Professor Jaeger, of Vienna, in 1861, began the first scientific examination of school children's eyes, according to Dr. Kaspar Pischl, of San Francisco, there have been over 200,000 pupils in all parts of the civilized world examined, and all those investigations have proven that myopia stands in a direct relation to the education of the people. This same writer has examined the eyes of 1,900 school children in the public schools of San Francisco, and his investigations show that myopia had increased from 3.98 per cent. in the grammar school to 11.59 per cent. in the normal school.

France Motais, in *Receuil d'Ophthalmologie*, after a careful examination of 5,000 students of schools and colleges in France, finds that myopia is acquired in 35 per cent. of the students of the most advanced classes. In the lowest classes he found none, in the intermediate 17 per cent., and in the advanced classes as stated above. He insists upon the extreme importance of adopting rigorous hygienic measures.

Professor Pfluger, in *Deutch Med. Zeitschrift*, has reiterated his complaint about the school system (in Switzerland), that myopia is exactly proportional to the number of hours of school work laid upon children, and quotes the results of Seggel's examination of 2,378 soldiers, that only 10 per cent. of the non-educated had myopia, while 60 per cent. of the student-soldiers were near-sighted.

Ramos-Vices, of Mexico, in an examination of 2,300 Mexican children of the lower schools, found myopia in only 0.3 per cent., while among 5,000 in the higher grades he found 19 per cent.

De Mets, of Antwerp, has examined over 7,000 school children with the following results: before the age of ten years 1.16 per cent. had myopia; after the age of ten years 3.10 per cent. had myopia. In well-lighted schools 1.63 per cent. of the scholars were found to be myopic, while in badly-lighted schools 3.73 per cent. of the scholars were myopic. He further says that bad light and increasing age, with increased demands made upon the eyes by study, are powerful factors in the development and augmentation of myopia.

Statistics from such accurate observers as Cohn in Germany, Sulzer in Switzerland, Haskett Derby in Boston, and Risley in Philadelphia, agree almost unanimously with those which have been given more in detail above. The statistics which have been given are taken from observers in different countries in order to show that this myopic increase is not confined to only one race of people.

If such then be the facts, is it not our duty as medical men to call the attention of the various boards which govern our public and private schools to these statistical facts, and endeavor as far as possible to make the school hygiene and regulations for the eyes as perfect as possible? The question of hygiene of the eyes has received far more attention in Europe than it has in our own country, but every day it is growing more in importance, and more attention is being given it by ophthalmologists all over the land.

Besides the question of myopia, or near-sightedness, to which all eyes tend, and which I have shown you increases among school children as they advance to higher grades, there is yet another phase of the subject which must demand as much sympathy and attention from us as does the question of myopia. It is the existence among school children of unsuspected, and therefore uncorrected, errors of refraction, especially low degrees of hyperopia and astigmatism, which retard greatly the physical and mental advancement of the individual and cause school life often to be one of pain and mental lassitude. This point has been very forcibly brought before the profession in an article by Dr. W. F. Southard, of San Francisco, read before the Ophthalmic Section at the last meeting of the American Medical Association, entitled "A Plea for the Examination of Every Child's Eyes when Commencing School," which was so timely throughout its extent that I shall take the liberty of presenting to you to-day some of the practical points therein contained.

Dr. Southard, in looking over the annual report of the public schools of San Francisco, found that there was an average daily attendance of 31,002 pupils between the ages of five and seventeen. Of this number 69.63 per cent. were in the primary grades, which included all children between the ages of five and ten years. 26.06 per cent. were pupils in the grammar grades, while but 4.31 per cent. were high school pupils. From these statistics Dr. S. calls attention to the fact that from the primary grades over one-half, or 50.43 per cent., fail to enter the grammar grades. At the close of the grammar grades 71.68 per cent., or more than three-fourths, fail to enter the high school. Is there then not a cause for surprise and alarm that only 4 per cent. of our primary pupils reach the high schools? Through inquiry from the superintendent of our public schools in Atlanta, I have ascertained that about 10 per cent. of the primary scholars enter the high school, a percentage somewhat higher than that found by Dr. Southard, but still a very insignificant number. That there must be some cause for this decrease is evident to all, and not only one but several factors by no means unimportant must enter into these results. It is true that when children complete the primary grades they are often taken from school in order to become wage-earners for the family, yet this number must be very small, since at the age of ten to twelve a child is not a very prominent wage-earning factor. Another factor which has been adduced, and to my mind the most prominent in this decrease, is the inability of a large number of children to keep up in their studies, as can be ascertained from teachers and superintendents who are daily brought in contact with these pupils.

That there is a uniform brain capacity and power of comprehension among children is tacitly recognized by all educators, since text-books, hours for study and gradation in schools have been arranged according to this idea. Why, then, is there not more uniformity in the results obtained, and why do we find so many children dropping out of school on account of their inability to keep up in their classes? If we inquire into the causes of this, we will find that ill health is the chief factor. Many children enter school when they are almost invalids; many are made to enter before they are old enough, developing the mind at the expense of the body; many are physically impoverished from bad hygienic surroundings and insufficient food at home; many have inherited weak constitutions, all of which interfere materially with their brain capacity.

In this very class if an examination was made we would often find a diminished acuity of vision. But besides the weak and sickly we often find scholars the brightest and the most healthy looking dropping out of the ranks of our public and private schools. As Dr. Southard says, for the first term or two they experience no difficulty, but later dropping behind they become discouraged. Such scholars are inattentive and listless, they seem to have lost the powers of concentration, and they are often called mischievous by their teachers and associates. They will complain frequently of headaches after and during school hours, or a dull heavy feeling in their eyes, and will be frequently corrected in the schoolroom for not paying attention. Teachers will complain to their parents in regard to these points, and one of the most frequent complaints is that the children will look on their books for a few moments and their eyes will wander around in a perfectly inattentive manner. The outcome of this is that teachers become impatient and petulant, scholars become disheartened and discouraged, while parents become dissatisfied and remove their children to another school, perhaps, only to have a reiteration of the old trouble. What then is the matter? If we examine such children we will frequently find that they are suffering with some unsuspected refractive error, such as I mentioned at the beginning of this paragraph. As an example of this, I recall one case most vividly: A young school girl, ten years old, had to be taken from school every year on account of incessant headaches, inability for close application for any length of time, and the consequent falling behind in her classes.

She was naturally a very bright child, and her parents could not divine the cause for all this trouble. On telling me this one day when she was in the office with a younger brother (who was being treated for adenoids), I examined her eyes and found three degrees of hypermetropic astigmatism. The correcting lenses were immediately prescribed with the happy result that the child now suffers with no headaches, is able to do her full amount of study, and as a result is standing among the first in the class.

Many poor children have been unnecessarily punished as mischievous and inattentive when, forsooth, they were unable to do their work on account of some existing error of refraction. Do not understand me as saying that such will be found in all cases. Far from it; but what I do mean is that refractive errors play no unimportant rôle. Besides these, must be mentioned poor light for schoolroom illumination, improperly constructed desks, bad ventilation, overcrowded seating capacity; all of which must add "fuel to the flame."

Having pointed out some of the errors which are liable to exist in the eyes of many school children, and which you must recognize from what I have said as potent factors in the well development of the mind and body, let us consider for a moment what steps should be taken to aid in the eradication of this existing evil. I fully agree with Dr. Southard in considering that the best remedy would be a compulsory examination of every child's eyes before entering school, and the presentation of a certificate to that effect just as is done with vaccination certificates. If this was done, and the proper corrections made, I believe that many a child would be freed from a life of mental and physical vicissitudes, school days would be happier and brighter, and the lot of a teacher would not be one of strife and vexation. Teachers should themselves receive proper instruction in regard to the hygiene of the eyes; they should be made acquainted with such symptoms as are recognized as a probable cause for the inattention on the part of the pupil, and should report it to the parents in order that a proper examination may be made and all refractive errors excluded. In some of our States lectures are delivered before the Normal Teachers' Association by some ophthalmologist. This is indeed a step in the right direction, which cannot but be productive of good.

Dr. Eaton, of Portland, Oregon, suggested at the last meeting of the American Medical Association, in the section of ophthalmology, that some steps should be taken by that section "to endeavor to have the various State medical societies formulate certain resolutions, so that the various legislatures, or at least the municipal authorities, might act upon the school-teachers in such a way for the benefit of the children" (as had been suggested by the various speakers).

In many of our schools the light is imperfect and badly placed, which often, with poor and badly printed text-books, is highly deleterious to the eyes. Then again the desks are sometimes badly constructed, so that frequently, to accommodate him or herself to the same, the pupil must distort the body, bend his head, cause congestion of the head and eyes, and thereby receive irreparable injury.

Should all existing errors of refraction in the eyes be corrected? That is a question which must be left to the judgment of every ophthalmologist for decision; but certain it is that in the majority of cases it is best to prescribe correcting lenses. Since statistics have shown that myopia or near-sightedness is on the increase as we ascend from the lower to the higher grades, and as myopia itself has a tendency to increase rather than diminish, should not those measures be adopted which will retard this increase? There is not one of you present to-day who would not answer that in the affirmative. What then are the proper measures to be taken in these cases? It is the proper correction of all unhygienic conditions of the schoolroom which exert an injurious influence upon the eyes; the correction of all errors of refraction by properly prescribed lenses, especially those of myopia. The beneficent effect of properly fitted lenses is well shown by Professor S. D. Risley, of Philadelphia, in a paper before the American Ophthalmological Society in May last, entitled "The Results of Treatment and the Optical Correction of Ametropia in Arresting the Increase of Myopia—A Clinical and Statistical Study." In this article Professor Risley has given us some important data, and the main one is this: That in the analysis of 200,000 eyes which had received glasses for optical correction of ametropia or defective vision, he found that there was a marked retardation in the progress of the ametropia; and especially was the treatment valuable in arresting the increase of myopia or near-sightedness. These statistics alone are sufficient to show the importance of the proper optical correction of all eyes suffering with defective vision.

To sum up in a more concrete form the main points brought forward in this article, we would say:

1. That the hygiene of school children's eyes should receive as much attention as that of any other portion of the body.

2. That in many cases where there is seeming physical ailment and mental inaptitude, such as headaches, dull feelings, inattention, loss of the power of concentration, the cause can be found in some ocular defect.

3. That as myopia or near-sightedness increases as the pupil advances to higher grades, such treatment should be instituted as will arrest the progress of that pathological condition.

4. That teachers should be instructed by competent ophthalmologists as to the hygiene of the eyes and the various symptoms which indicate some ocular defect.

5. That every child should have his or her eyes examined before entering school, and if necessary, a certificate should be given to that effect.

*The Grand.*

